# Cluster headache

**DOI:** 10.1186/1750-1172-3-20

**Published:** 2008-07-23

**Authors:** Elizabeth Leroux, Anne Ducros

**Affiliations:** 1 Centre d'Urgences Céphalées, Hôpital Lariboisière, Paris, France

## Abstract

Cluster headache (CH) is a primary headache disease characterized by recurrent short-lasting attacks (15 to 180 minutes) of excruciating unilateral periorbital pain accompanied by ipsilateral autonomic signs (lacrimation, nasal congestion, ptosis, miosis, lid edema, redness of the eye). It affects young adults, predominantly males. Prevalence is estimated at 0.5–1.0/1,000. CH has a circannual and circadian periodicity, attacks being clustered (hence the name) in bouts that can occur during specific months of the year. Alcohol is the only dietary trigger of CH, strong odors (mainly solvents and cigarette smoke) and napping may also trigger CH attacks. During bouts, attacks may happen at precise hours, especially during the night. During the attacks, patients tend to be restless. CH may be episodic or chronic, depending on the presence of remission periods. CH is associated with trigeminovascular activation and neuroendocrine and vegetative disturbances, however, the precise cautive mechanisms remain unknown. Involvement of the hypothalamus (a structure regulating endocrine function and sleep-wake rhythms) has been confirmed, explaining, at least in part, the cyclic aspects of CH. The disease is familial in about 10% of cases. Genetic factors play a role in CH susceptibility, and a causative role has been suggested for the hypocretin receptor gene. Diagnosis is clinical. Differential diagnoses include other primary headache diseases such as migraine, paroxysmal hemicrania and SUNCT syndrome. At present, there is no curative treatment. There are efficient treatments to shorten the painful attacks (acute treatments) and to reduce the number of daily attacks (prophylactic treatments). Acute treatment is based on subcutaneous administration of sumatriptan and high-flow oxygen. Verapamil, lithium, methysergide, prednisone, greater occipital nerve blocks and topiramate may be used for prophylaxis. In refractory cases, deep-brain stimulation of the hypothalamus and greater occipital nerve stimulators have been tried in experimental settings. The disease course over a lifetime is unpredictable. Some patients have only one period of attacks, while in others the disease evolves from episodic to chronic form.

## Disease name and synonyms

Other terms for cluster headache (CH) are erythroprosopalgia of Bing, ciliary or migrainous neuralgia, erythromelalgia of the head, Horton's headache, histaminic cephalalgia, petrosal neuralgia of Gardner, sphenopalatine, Vidian and Sluder's neuralgia, and hemicrania periodica neuralgiformis. In French, it is named *algie vasculaire de la face*, somewhat a misnomer since CH does not primarily involve a dysfunction of arteries or veins.

## Definition, diagnostic criteria and forms

Cluster headache is a primary headache disease like migraine or tension-type headache. By contrast to secondary headaches, in which the pain is symptomatic of an underlying brain, cranial or systemic disorder, primary headaches are due to spontaneous activation of nociceptive pathways. CH is characterized by recurrent attacks of short-lasting excruciating pain accompanied by signs of autonomic dysfunction, as described in the second version of the International Classification Headaches second edition (ICHD-II) criteria presented in table 1[1]. The patient must have had at least five attacks of severe or very severe unilateral orbital or supraorbital and/or temporal pain, lasting 15 to 180 minutes if untreated. The headache is accompanied by one ipsilateral autonomic symptom among the following: conjunctival injection and lacrimation, nasal congestion or rhinorrhea, forehead and facial sweating, eyelid edema, miosis and ptosis. In the absence of autonomic signs, CH can be diagnosed if a sense of restlessness or agitation is present during the attacks.

**Table 1:** Diagnostic criteria for cluster headache according to the International Classification of Headache Diseases II

**3.1 Cluster headache**
A. At least five attacks fulfilling B through D
B. Severe or very severe unilateral orbital, supraorbital and/or temporal pain lasting 15 to 180 minutes if untreated
C. Headache is accompanied by at least one of the following:
1. Ipsilateral conjunctival injection and/or lacrimation
2. Ipsilateral nasal congestion and/or rhinorrhea
3. Ipsilateral eyelid edema
4. Ipsilateral forehead and facial sweating
5. Ipsilateral miosis and/or ptosis
6. A sense of restlessness or agitation
D. Attacks have a frequency from one every other day to eight per day
E. Not attributed to another disorder

CH is the most common type of the trigeminal autonomic cephalalgias (TACs), which include paroxysmal hemicrania and SUNCT (**S**hort **U**nilateral Neuralgiform headache with **C**onjunctival injection and **T**earing). It is divided in episodic (3.1.1) and chronic (3.1.2) forms [1], as detailed in table 2. In 80% of cases, the disease is episodic and may present with one or two periods per year, or even may go into remission for many years before development of another cluster period. In about 20% of CH patients the disease is chronic [2]. These patients have ongoing attacks for one year or more, without more than one month of remission. These refractory patients are difficult to diagnose and manage, and need to be referred to a headache specialist.

**Table 2:** ICHD-II criteria for episodic and chronic cluster headache

**3.1.1 Episodic cluster headache**
A. All fulfilling criteria A through E of 3.1
B. At least two cluster periods lasting from 7 to 365 days and separated by pain free remissions of > 1 month.

**3.1.2 Chronic cluster headache**
A. All fulfilling criteria A through E of 3.1
B. Attacks recur for > 1 year without remission periods or with remission periods lasting < 1 month.

## Epidemiology

The prevalence of CH can be estimated at approximately 0,5 – 1.0/1,000 [3-12]. Table 3 summarizes the epidemiological studies on CH. Many of these studies are biased by the sample chosen and by the difficulties of diagnosing CH by mailed questionnaires or telephone interviews. In a tertiary care headache clinic, CH was diagnosed in 2.73% of patients. There is a clear male predominance in CH, but it has been estimated to be decreasing over the years [13], maybe due to increased recognition of the disease in women [14]. CH is a condition affecting young adults, first attacks usually occurring in the third decade. It is probably under-diagnosed and unrecognized; a significant diagnostic delay has been reported in most patients [15,16]. CH in children is rare, but a few cases have been reported [17], the youngest patients being three years old [18]. Symptoms often decrease after age of 70 years. Heavy smoking has been frequently documented amongst CH patients [19].

**Table 3:** Epidemiological studies of cluster headache

Country	Diagnosis confirmed	Age	Sex	Population sample	Affected	Prevalence per 100 000
Sweden [10]	Yes	18	Men	9803	9	92
San Marino [3]	Yes	All	Both	21792	14	69
USA [6]	No	All	Both	6476	26	401
San Marino [7]	Yes	All	Both	26628	15	56
Norway [5]	Yes	18–65	Both	1838	7	381
Sweden [11]	Yes	All	Both	31750	48	151
Italy [8]	Yes	18–65	Both	6500	13	200
Italy [9]	Yes	Over 14	Both	10071	21	279
Germany [4]	Yes	18–65	Both	3336	4	119
Germany [12]	Yes	25–75	Both	2291	2	150

## Clinical description: how to recognize cluster headache

### Duration and frequency of attacks

Cluster headache attacks are unilateral, severe, short-lasting (15 to 180 minutes) and recurrent (1 every other day to 8 per day) [1]. This special pattern is very important to recognize the cluster headaches and to differentiate them from migraine attacks. A patient describing a pain lasting less than three hours, severe and localized on one side of the head, coming back at specific times of the day or during sleep, must be asked if symptoms like tearing, eye swelling and nasal congestion are present on the other side. The fact that the attack ends after one or two hours must not be attributed to a success of standard analgesics, which are not useful in CH.

### Pain description

A prospective study on clinical characteristics of CH has been published by Bahra *et al*. [2]. Pain is described mostly in the first trigeminal branch territory, on one side of the head only and almost always the same side (rarely, attacks may switch the location). Pain may be centered on the eye but may also be periorbital and temporal, sometimes extending to the maxillary, ear or even occipito-cervical region. Some patients describe the pain as radiating to the teeth, thus they may first consult a dentist. As to its severity, CH is one of the most painful conditions known to man (worse than giving birth according to female patients). It is described as excruciating and intolerable. Patients compare it to a white-hot needle or knife being pushed into the eye, the eye being crushed or ripped of the orbit. Suicidal ideations may be expressed during the attacks. The nickname of the CH among patients is "The Beast", a term suggesting an animal hiding in the shadow, attacking in a terrible and unpredictable way. Pain rises abruptly over a few minutes. It also ends rapidly. In severe chronic forms or in episodic forms with numerous daily attacks, there may be a continuous discomfort on the affected side [20], but usually CH patients are completely asymptomatic between attacks.

### Dysautonomic symptoms

Parasympathetic hyperactivity signs include ipsilateral lacrimation, redness of the eye and nasal congestion. The affected side of the face may be red and sweaty. Sympathetic hypoactivity is demonstrated by the combination of ipsilateral ptosis and miosis (droopy eye and smaller pupil on the affected side) during attacks. The combination of ptosis and miosis on one side is called Claude-Bernard-Horner syndrome (CBH). Persistence of CBH signs after the end of the attack in episodic CH should alert the clinician to suspect underlying lesion in hypothalamus, brainstem, paravertebral sympathetic chain or carotid. Proper investigations should be undertaken: brain magnetic resonance imaging (MRI), chest computerized tomography and carotid ultrasound.

Autonomic signs are very prominent in certain patients, but may be subtle or even absent in 3% of patients [21]. They can be unrecognized because of the pain ("I close my eye, I cry because I'm in pain"). In case of uncertainty, the patient can be asked to observe these specific signs during the attack.

### Ictal behavior

Physical activity seems to partially alleviate the pain in CH. During the attacks, patients tend to be restless. They may rock from side to side, hit their heads, hit objects with the fist, or even hit their head against the wall [22]. This behavior is so typical of CH that it is accepted as an official criterion in the ICHD-II [1]. Indeed, it is possible to diagnose CH without any dysautonomic sign if the behavioral criterion is satisfied. This restless attitude may help to differentiate CH from migraine, where patients are quiet and avoid any movement. CH patients are cognitively alert, but may also be irritable and aggressive. There is no mental slowing or fatigue, as it may be seen in migraine patients.

### Cyclicity

CH shows circannual and circadian cyclicity. Peaks have been described around solstices, in relation to changes in daylight duration [23]. Attacks can happen at a precise time during the sleeping cycle, with dramatic regularity reported by patients. This cyclicity is not observed in all CH patients and is not included in the ICHD-II criteria, though most experts find it very suggestive of CH when present.

### Triggers

Alcohol is recognized as the only dietary trigger of CH. Nitroglycerin has been used to trigger attacks for diagnostic purpose (therefore, e nitrates should be avoided during bouts). Strong odors (mainly solvents and cigarette smoke) and napping may trigger CH attacks. It is surprising that all triggers are effective only during bouts. In between periods, patients may smoke and drink alcohol without triggering attacks.

### Diagnostic delay

There is a significant delay in diagnosing CH (reported median delay of three years [16]), though there is an improvement as compared to the 1960's when a diagnostic delay of more than 20 years was not infrequent [15]. Up to 30% of patients have previously undergone many investigations and seen dentists, ENT (ears, nose, throat) surgeons, ophthalmologists, even neurologists, without being diagnosed. Up to 16% may have undergone dental, sinus or eye surgery [16]. The periodicity of attacks makes the diagnosis difficult and sometimes suggests an infectious cause. Prednisone can be prescribed for sinusitis and have a positive effect, falsely confirming an erroneous diagnosis. Some patients do not seek medical advice once the bout has ended by itself.

### Other features

Though aura is not frequent in CH [24], up to 14% of patients report aura symptoms, with transient visual, motor or sensory disturbances preceding the facial pain [2]. In chronic CH, auras have been reported in 20% of patients [20]. Nausea, vomiting, photophobia and phonophobia can be observed in approximately 50% of patients, and must not discard the diagnosis of CH if other criteria are fulfilled. A shift of the affected side may be observed within a bout or between two bouts in 15% of patients [2]. Case reports of "CH sine headache" have described patients with episodes of unilateral facial autonomic dysfunction without pain [25,26], but this is very rare.

## Etiology: a role for the hypothalamus, but many uncertainties

The exact cause of CH is currently unknown. The first hypotheses on CH, inspired by the effects of vasoactive substances in CH (dilators causing attacks, constrictors ending them) were based on the neurovascular theory. An initial dysfunction or inflammation of the blood vessels in the parasellar or cavernous sinus area would activate the orbital trigeminal pain pathways. Indeed, activation of the trigeminal-vascular system is present, but whether it is a cause or consequence of CH is not clear. It is not specific for CH, since it is also very prominent, and much better demonstrated, in migraine. Later on, the periodicity of CH has oriented studies toward the hypothalamus, with more fruitful results. Positron emission tomography (PET) studies have show an activation of the posterior hypothalamus during CH attacks [27], reinforcing the idea that the hypothalamus may be the CH generator. Voxel-based morphometry studies demonstrated an increase in the gray matter volume of the posterior hypothalamus in CH patients compared to controls [28]. Success of hypothalamic deep-brain stimulation (DBS) [29] and emerging evidence for hypocretin receptor gene involvement also strengthen that hypothesis. Abnormal blood levels of prolactin, testosterone, thyroid stimulating hormone (TSH), and cortisol may be linked to perturbations in the activity of the hypothalamic-pituitary-adrenal axis.

Numerous studies have examined blood levels of other substances in CH patients during attacks, bouts and remissions. Histamine, prostaglandins, opiates, neuropeptids, amines, nitric oxide, monoamine oxydase, serotonin and cytokines values may show significant differences compared to those in non-affected controls. The list is long but, so far, none of these studies allowed a coherent understanding of CH. The parasympathetic activation is thought to be mediated by the trigeminal-autonomic reflex in the brainstem circuitry,*via *the seventh cranial nerve. The sympathetic innervation pathway to the pupil and eyelid is composed of three neurons and there is no clear demonstration of the site involved and mechanisms underlying the dysfunction in CH. The reason for the male predominance of CH also remains to be clarified.

### Genetics

Initially, CH was not thought to be a genetic disease. With official criteria published and the increasing recognition of the disease, a genetic aspect of CH has been identified. Cases of twin pairs affected by CH have been published [30-32]. Studies using large twin registries have shown that monozygotic twins present a higher concordance rate for CH (2/12 pairs) than dizygotic twins (0/25 pairs), indicating the existence of genetic factors, but monozygotic twins can also be concordant or discordant for CH, confirming a role for environmental factors [11,33]. Family studies of CH patients' cohorts have shown a familial aggregation in 7% to 20% of patients, and a relative risk for first-degree relatives between 14 and 39 [34-36]. The transmission mode has still not been established. Some studies have suggested an autosomal dominant way with incomplete penetrance [37], while other described an autosomal recessive transmission [38]. So far, no X-linked pedigrees have been published. No susceptibility locus has been identified. Much work has recently been done on the *HCRTR2 *gene (hypocretin receptor 2) polymorphism [39,40], suggesting a role of this gene in CH susceptibility. Hypocretins are secreted in the hypothalamus, a structure clearly involved in CH. All these findings suggest that genetic factors do play a role in CH [41,42], though, so far, the transmission mode remains to be established and the contribution of individual factors to be clearly understood. The *HCRTR2 *gene is a promising research area.

## Diagnostic methods

Diagnosis of cluster headache is based on clinical criteria and exclusion of a secondary cause. A first attack suggestive of CH must always be thoroughly investigated, and carotid dissection has to be properly excluded either with doppler ultrasound or angio-scanner and magnetic resonance angiography of the neck vessels [43]. When the history is typical with numerous periods, attacks and no interictal abnormalities on neurological examination, MRI is not mandatory. Imaging studies in CH patients are most of the time normal, but lesions can be detected in secondary cases. An association with pituitary tumors has been suspected [44]. Any refractory case should undergo MRI study to exclude a treatable cause. The pituitary, orbit, and trigeminal pathway have to be specifically examined with computed tomography (CT) and MRI.

## Differential diagnosis

### Primary headache diseases

#### Migraine

The only way to differentiate CH from migraine is the clinical history. The questionnaire is of paramount importance. The global picture must be taken into account. In case of doubt, further observation of attacks by the patient is warranted. Migraine and CH are both very incapacitating and share common features, but they also differ on specific points. Migraine is much more frequent than CH (15% of the general population versus 0.05%–0.1%), affects mainly women, and starts earlier in life, often during adolescence or around menarche. Migraine attacks occur at regular intervals over months, without long remission periods, though in some patients seasonal triggers may cause more frequent attacks (allergies, stress). Migraine attacks last longer (more than four hours and can go on for more than 24 hours) [2]. Attacks may have a prodrome with hunger, fatigue, irritability, and start more progressively. Patients tend to stay quiet and avoid any movement. The pain may begin unilaterally but during the attack it may radiate to the whole head. Many patients have attacks on alternate sides. Pain is moderate-to-severe, but is not described as intolerable or excruciating. The presence of nausea and/or photophobia and phonophobia is a main clinical criterium [2], but it is not specific for migraine, as CH can also be accompanied by nausea, photophobia and phonophobia in up to 50% of patients [2]. Unilateral photo- and phonophobia may be more specific of CH and trigeminal autonomic cephalalgias in general [45]. In up to 30% of patients with migraine, autonomic signs like lacrimation and nasal congestion are present. Both CH and migraine can be triggered by alcohol, and relieved by triptans, but CH necessitates parenteral routes of administration. Stress, foods (like chocolate) and menstrual cycle, are not typical triggers for CH. Finally, some cases are difficult to diagnose and should be referred to a headache specialist. In some patients, CH and migraine can coexist, and treatment must be chosen accordingly. Table 4 presents a clinical comparison of CH and migraine.

**Table 4:** Comparison of migraine and cluster headache

Migraine	Cluster headache	Common characteristics
No periodicity (except with menses)	Periodicity (annual and daily)	Incapacitating
		Alcohol is a trigger
Attacks > 4 h	Attacks < 3 h	Triptan effect (spray and subcutaneous)
Female > Male	Male > Female	
Prostration, quietness	Restlessness, agitation	Dysautonomic symptoms (typical in CH, but may occur in migraine)
Pain moderate to severe	Pain is severe	
Pain can be bilateral	Unilateral	
Nausea and photophobia typical	Nausea and photophobia can happen but not typical	
Dietary and hormonal triggers	No dietary trigger except alcohol	NB The two types of headache may coexist

#### Other trigeminal autonomic cephalalgias (TACs)

Paroxysmal hemicrania (PH) is more frequent in women (80–90%) and differs from CH by the shorter length and higher frequency of attacks [46-48]. Absolute response to an adequate dose of indomethacin (150 mg per day or more) confirm the diagnosis of PH [2]. A continuum between paroxysmal hemicrania and CH has been suggested by some authors, due to an overlap in clinical criteria and anecdotal reports of CH patients responding to indomethacin. Indomethacin treatment in any refractory case of CH, or cases with frequent and shorter attacks, has been suggested by Matharu and Goatsby [49].

SUNCT syndrome is a very rare entity [50]. Its prevalence is estimated around 6,6 per 100,000 [51]. Attacks are very short and occur up to 300 times a day.

Hemicrania continua may be confused with chronic cluster headache, and if there is a doubt, a trial with indomethacin should be made, HC being highly responsive to this drug [52,53]. Table 5 presents the main characteristics of TACs.

**Table 5:** Trigeminal-autonomic cephalalgias (TACs)

	**Cluster headache**	**Paroxysmal hemicrania**	**SUNCT**
Sex ratio M: F	3: 1	1: 2	2: 1

Duration of attacks	15–180 min	2–30 min	5–240 sec
Frequency of attacks	1/2 days to 8/day	5–40/day	3–200/day
Periodicity	Episodic form may be seasonal	Episodic or chronic	Episodic or chronic

Treatment	Verapamil	Indomethacin	Anticonvulsants
	Lithium		
	Steroids		

#### Other ICHD-II categories

Trigeminal neuralgia (TN) and CH both involve paroxysms of unilateral and severe pain, but specific traits are found on the questionnaire [2]. TN is more frequent in women in their fifties or older. Attacks are centered on maxillary and/or mandibular area, they are more frequent, briefer, electric-like and are triggered by touching specific zones of the face or buccal cavity. Eating, laughing, talking, shaving or brushing the teeth may all trigger the shocks. Patients may loose weight because they avoid eating. There is no clustering in periods, and mostly the pain goes on until the treatment starts. TN responds very well to carbamazepine. Both diseases may occur in the same patient as cluster-tic (tic standing for tic douloureux, a French term). Table 6 summarizes the clinical manifestations helping to distinguish TN from CH.

**Table 6:** Comparison of cluster headache and trigeminal neuralgia

	**Cluster headache**	**Trigeminal neuralgia**
Age	Starts around 20–30	60
Sex	Male	Female
Pain localization	Orbital, temporal	Nasal, maxillary, dental
Attack duration	15 to 180 minutes	Seconds, but repeated shocks
Pain character	Knife-like, stabbing, lancinating	Electric shocks, burning, stinging
Trigger zone	No	Yes
Neurovegetative signs	Yes	No
Refractory period after attack	Not typical	Yes
Frequency per day	1–8	Highly variable but may be dozens or more

In an elderly patient with exclusively nocturnal attacks, hypnic headache may be diagnosed, but it is very rare and pain is usually bilateral, diffuse and without autonomic signs. Attacks occur exclusively during sleep [54].

In doubt, the clinician may ask the patient to complete a diary of the attacks, to precise their duration, autonomic symptoms, triggers, response to treatment, and daily cyclicity. The importance of history taking in headache medicine cannot be overemphasized.

### Causes of symptomatic CH

Features suggesting a secondary CH are: persistent or worsening pain, abnormal signs on neurological examination, and a first period after 50 years old. An underlying cause has sometimes been found in a patient with typical CH features [55,56]. Benign intracranial tumors like meningiomas and neurinomas, but also carcinomas and metastases, have been reported. There seems to be an overrepresentation of pituitary tumors in CH patients [44], and a role of prolactin has been suggested in these cases. Orbital tumors or infections have been reported. Sinusitis may present with symptoms typical for CH. Arteriovenous malformations and aneurysms, if strategically located, may cause CH symptoms. In a first episode, carotid dissection has to be suspected and ruled out (it also can produce Claude-Bernard-Horner syndrome, and the pain may respond to triptans) [57]. Any case of refractory CH deserves a brain MRI. Some experts even recommend MRI for every CH patient, to avoid unnecessary treatments if a treatable cause is found.

## Genetic counseling

As familial cluster headache is rare, thus, considering the limited knowledge on the genes involved and the transmission mode, genetic counseling is not routinely part of the CH management. Familial cases should be identified and, if possible, the family referred to a specialist in cluster headache. There is no known preventive treatment for CH, or curative intervention, but affected patients may inform their families of the CH symptoms, thus any delay in diagnosis among relatives to be avoided. Antenatal diagnosis is not applicable

## Management

Acute treatments are efficient to alleviate pain during attacks. Prophylactic treatments aim at reducing the daily number of attacks. No curative treatment for CH is currently available. Guidelines (evidence-based recommendations and good practice points) for the treatment of CH are developed by the European Federation of Neurological Societies (EFNS) [58].

### Acute management of attacks

Because of the excruciating character of the pain, rapid-acting treatments are preferred for acute management of attacks [59]. The oral route of administration is too slow and is rarely used. Inhaled, subcutaneous and intra-nasal treatments are preferred. The pain in CH is often underestimated by the medical community. Interictally, patients appear normal. In most cases, witnessing a CH attack is convincing enough, but not possible for every practitioner. Adequate treatments must be provided, and a nihilistic, observational approach should be absolutely avoided.

The two most efficient acute treatments are subcutaneous sumatriptan and high-flow inhaled oxygen.

Sumatriptan is a serotonin 5HT1 B/D agonist, acting on blood vessels with a vasoconstricting effect, and centrally on brainstem receptors. Subcutaneous sumatriptan 6 mg (Imiject, Imitrex) has been shown to efficiently relieve the CH pain and dramatically improve the management of patients [60]. After the injection, the patient may feel a rush of heat and chest tightness that disappear in a few minutes and are followed by rapid relief of the pain. Intra-nasal sumatriptan [61,62] and zolmitriptan [63] may be tried if patients refuse to use injections. Absolute contra-indications to subcutaneous sumatriptan include pregnancy, lactation, coronary artery disease, stroke, and peripheral artery disease. Relative contra-indications are age (under 18 or over 65), an isolated controlled vascular risk factor, Raynaud phenomenon, allergy to sulfa medications, treatment with a serotonin recapture inhibitor (SSRI). Maximal doses for 24 h period (sumatriptan 12 mg subcutaneous or 40 mg intra-nasal, zolmitriptan 10 mg intra-nasal) have to be explained to the patient to avoid dangerous side effects, such as coronary or distal artery spasm. Surprisingly, CH patients do not develop medication-overuse headache (MOH) with triptans as often as migrainers do [64]. They may use regular sumatriptan for many months without developing a different type of headache. As MOH is more frequent in CH patients with a history/diagnosis of migraine, these patients must be carefully monitored for MOH, especially if CH is chronic and refractory [65].

Inhaled oxygen with high-flow (10 to 15 l per minute) 100% normobaric oxygen breathed through a mask is efficient and useful especially when contra-indications to triptans exist [66,67]. The device is not easy to carry around, and not always reimbursed by health security services, depending on the country. The efficacy of oxygen has been recently re-examined and confirmed. There are no contra-indications or limitations for the use of oxygen.

Ergotamine and dihydroergotamine have been used in CH [68]. They must not be combined with triptans on the same 24 hour period, have the same contra-indications as triptans (without additional efficacy), therefore are rarely useful.

Intra-nasal application of topical lidocaine may be tried in refractory CH cases or in case of contra-indications to triptans. Its efficacy is not well proven.

Topic cocaine was used in the past, but abandoned because of equivocal efficacy, side-effects and risks of dependency.

### Prophylactic treatment

The aim of prophylactic treatment is to reduce the frequency of attacks during the bout. Continuous use of prophylactic treatments in episodic CH is not recommended, since there is no proof that it prevents the next bout. Therefore, the dose may be progressively decreased after two or three months, or after the usual known duration of the bout. The prophylactic treatment should be restarted if attacks reappear. In patients with few attacks per week responding to acute treatment, prophylactic treatment can be withheld. In chronic CH, treatments are used for a long term epriod, and a low attack rate has to be reached (always considering the potentially serious and incapacitating side effects).

The calcium-channel blocker verapamil remains the main treatment of episodic and chronic CH [69,70]. Doses range from 360 mg to 960 mg per day, and the maximal tolerated dose should be reached before concluding about efficacy. Side effects are weakness, fatigue, lower extremities edema, and conduction heart block. An electrocardiogram should be performed at the baseline and for every increase in the dose when it exceeds 480 mg per day [71].

Greater occipital nerve blocks are used, based on the connection between this nerve and the trigeminal circuitry [72]. One randomized study showed that this technique may end a bout, or, at least, may diminish the number of attacks per day [73]. More evidence is needed to recommend this procedure [74], but it is widely used by experts [75,76]. The block is performed with a long-acting injectable steroid, sometimes combined with a local anesthetic. Different combinations have been described. The needle is inserted midway between the mastoid and the occipital protuberance, until it touches the occipital bone (sub occipital technique).

Topiramate has shown a convincing efficacy in CH [77,78]. Usual dosing is 100 mg per day (range 25 mg to 200 mg). Side effects include numbness and tingling in the extremities, weight loss, and neuro-cognitive difficulties, all reversible after drug discontinuation.

Lithium is used in chronic CH [69,79,80]. Doses range from 600 to 1200 mg per day. Blood levels must not exceed 0,9 mEq per liter, and thyroid and renal function must be monitored. Side effects include tremors, insomnia, fatigue, nausea and blurred vision. Intoxication may be severe and has to be suspected quickly if nausea, confusion, rigidity and difficulty to walk appear. Because of these side effects, lithium is used sparingly, in selected cases only.

A short course of decreasing-dose prednisone (starting at 1 mg/kg) may be used to treat a bout refractory to verapamil (however, recurrence is frequent with the decrease of the dose). Long-term side effects of corticotherapy restrict the use of prednisone in chronic CH.

Methysergide acts on serotonin receptors. Doses range from 6 to 12 mg per day. Some patients do respond very well to this drug but a major limitation is that triptans and ergots cannot be used simultaneously because of the risk of serotoninergic syndrome. The main side effect is nausea, sometimes severe. Methysergide can be considered if oxygen alone is used as an acute treatment. The main complication is retroperitoneal, pulmonary or cardiac fibrosis after prolonged use. This mandates one-month drug-holiday after six months of use, then methysergide can be started again. Methysergide is not available in United States.

Valproic acid [81], pizotifen, gabapentin [82,83], baclofen [84], and melatonin [85,86] have shown some effect in CH prophylaxis and may be used in selected patients as a second-line therapy.

### Surgical approaches

Destructive approaches like trigeminal section, thermocoagulation of gasserian ganglion, glycerol rhizotomy [87,88] and radiosurgery [89-92] of the trigeminal nerve have shown variable results and irreversible complications like anesthesia dolorosa.

Hypothalamic deep-brain stimulation (hDBS) has shown a convincing benefit in some refractory patients in series totalizing a few dozens of cases [93-96], but can be completely inefficient[97], and has potential serious complications. Its mechanism is not well understood[98] and no controlled studies are available.

Neurostimulation of the greater occipital nerve has recently been described [76,99-101]. It is less invasive than DBS but costly; its long-term effects are unknown.

Non-pharmacological approaches like massotherapy, physiotherapy and acupuncture have not been adequately studied in CH.

## Prognosis

CH has a very unpredictable course. Some patients have only one period of attacks, while in others the disease evolves from episodic to chronic form. There are no known factors for chronicization of the CH. Total remission of the disease has been described. With aging, attacks often decrease and active CH is rarely seen after age of 75 years.

## Conclusion

CH is a primary trigeminal-autonomic cephalalgia responsible for recurrent unilateral short-lasting attacks of excruciating orbitotemporal pain. The cause of cluster headache is not known and there is no unifying hypothesis to explain all the clinical particularities of this disease. About 10% of CH patients have a familial form and the existence of genetic factors has been confirmed. The hypocretin receptor gene may be involved, but implication of other genes can't be excluded. In the absence of any radiological or biological marker, diagnosis relies upon clinical history. Patients are often misdiagnosed as having secondary headaches due to sinus, dental or eye disorders or as having migraine, leading to inadequate management. No curative treatment currently exists, however efficient treatment do exist to shorten painful attacks (acute treatments) and to reduce the number of daily attacks (prophylactic treatments). Ongoing research is very active. Deep-brain stimulation and occipital nerve stimulators are promising options for refractory patients but are used currently in an experimental setting. The current pathophysiological focus is on the hypothalamus. The combination of imaging techniques and genetic studies may help us to better understand this terrible and fascinating condition, ultimately leading to improvement of the care and quality of life of these patients.

## Abbreviations

CH: cluster headache; ICHD-II: International Classification Headaches second edition; TAC: trigeminal-autonomic cephalalgia; SUNCT: Short Unilateral Neuralgiform headache with Conjunctival injection and Tearing; CBH: Claude-Bernard-Horner syndrome; *HCRTR2 *gene: hypocretin receptor 2 gene; MRI: magnetic resonance imaging; ENT: ears, nose, throat; PET: positron emission tomography; hDBS: hypothalamic deep-brain stimulation; TSH: thyroid stimulating hormone; PH: paroxysmal hemicrania; EFNS: European Federation of Neurological Societies; SSRI: serotonin recapture inhibitor; MOH: medication-overuse headache.

## Competing interests

The authors declare that they have no competing interests.

## Authors' contributions

The two authors equally contributed to this review article. They read and approved the final version of the manuscript.
